# Hedgehog Inhibitors Suppress Osteoclastogenesis in In Vitro Cultures, and Deletion of *Smo* in Macrophage/Osteoclast Lineage Prevents Age-Related Bone Loss

**DOI:** 10.3390/ijms21082745

**Published:** 2020-04-15

**Authors:** Yukihiro Kohara, Ryuma Haraguchi, Riko Kitazawa, Yuuki Imai, Sohei Kitazawa

**Affiliations:** 1 Department of Molecular Pathology, Ehime University Graduate School of Medicine, Shitsukawa, Toon City, Ehime 791-0295, Japan; 2 Division of Diagnostic Pathology, Ehime University Hospital, Shitsukawa, Toon City, Ehime 791-0295, Japan; 3 Division of Integrative Pathophysiology, Proteo-Science Center, Ehime University Graduate School of Medicine, Shitsukawa, Toon City, Ehime 791-0295, Japan; 4 Division of Laboratory Animal Research, Advanced Research Support Center, Ehime University, Toon, Ehime 791-0295, Japan; 5 Department of Pathophysiology, Ehime University Graduate School of Medicine, Toon, Ehime 791-0295, Japan

**Keywords:** osteoclasts, Hedgehog signaling, cyclopamine, GANT-58, GANT-61, Smoothened, GLI1, GLI2

## Abstract

The functional role of the Hedgehog (Hh)-signaling pathway has been widely investigated in bone physiology/development. Previous studies have, however, focused primarily on *Hh* functions in bone formation, while its roles in bone resorption have not been fully elucidated. Here, we found that cyclopamine (smoothened (Smo) inhibitor), GANT-58 (GLI1 inhibitor), or GANT-61 (GLI1/2 inhibitor) significantly inhibited RANKL-induced osteoclast differentiation of bone marrow-derived macrophages. Although the inhibitory effects were exerted by cyclopamine or GANT-61 treatment during 0–48 h (early stage of osteoclast differentiation) or 48–96 h (late stage of osteoclast differentiation) after RANKL stimulation, GANT-58 suppressed osteoclast formation only during the early stage. These results suggest that the Smo-GLI1/2 axis mediates the whole process of osteoclastogenesis and that GLI1 activation is requisite only during early cellular events of osteoclastogenesis. Additionally, macrophage/osteoclast-specific deletion of Smo in mice was found to attenuate the aging phenotype characterized by trabecular low bone mass, suggesting that blockage of the Hh-signaling pathway in the osteoclast lineage plays a protective role against age-related bone loss. Our findings reveal a specific role of the Hh-signaling pathway in bone resorption and highlight that its inhibitors show potential as therapeutic agents that block osteoclast formation in the treatment of senile osteoporosis.

## 1. Introduction

Hedgehog (Hh) signaling, well-known as a mitogen and morphogen during animal development, also regulates adult tissue homeostasis and tumorigenesis [1,2,3,4]. Hh ligands in mammals are three proteins, Indian hedgehog (Ihh), Sonic hedgehog, and Desert hedgehog; although they are expressed in different cells and tissues, their functions are not considered different [5,6]. Hh ligands bind to and inhibit main receptor Patched-1 (PTCH1, encoded by the *Ptch1* gene), permitting the activation of Hh signal transducer smoothened (SMO, encoded by the *Smo* gene) and transmitting intracellular signaling through transcription factors of the GLI family [5,7,8]. Among GLI transcription factors GLI1, GLI2, and GLI3 that collectively mediate all Hh pathways, GLI2 and GLI3 are the initial mediators of Hh signal transduction, and GLI1, being a direct target gene, functions as a positive feedback to enhance GLI activity [8]. GLI1 acts as a positive transcriptional effector, while GLI2 and GLI3 function predominantly as transcriptional activators or repressors in a cellular context-dependent manner. In the activated Hh-signaling pathway, GLI proteins are released from the inhibitory complex with the Suppressor of Fused (SuFu) [9,10]. Finally, activated GLI forms are translocated to the nucleus, where they act as transcription factors and promote Hh target gene expression.

Agents that specifically and selectively target the Hh-signaling pathway are available for experiments [11,12,13,14,15,16]. Cyclopamine is a bioactive steroidal alkaloid extracted from natural plants, and its synthetic compounds inhibit SMO function by direct interaction with SMO-transmembrane domains [14,15]. GANT-58 and GANT-61 are identified as small-molecule inhibitors of GLI proteins [11,13]. GANT-58 prevents GLI1-dependent transcription through the inhibition of its post-translational modification [11]. In contrast, GANT61 blocks GLI1/DNA interaction by direct binding to the GLI1 protein and impairs GLI2-mediated transcription [11,13]. The GANT61-binding element shows a high degree of sequence homology between GLI1 and GLI2, making GANT61 an inhibitor of both GLI1- and GLI2-induced transcriptions [13]. At present, targeting Hh signaling by inhibitors, including cyclopamine and GANTs, has been drawing attention as a potential therapeutic strategy in various human diseases.

The Hh-signaling pathway contributes to skeletal development, bone homeostasis, and the progression of tumor bone metastasis. During endochondral ossification, Ihh produced by hypertrophic chondrocytes stimulates osteoblastic bone formation by promoting the expression of *Runx2,* which is known as a master transcription factor for osteoblast differentiation [2,17,18]. The study of Rodda and McMahon revealed that Hh signaling is not required in the early differentiation phase of an osteoblast for further osteoblast maturation [19]. In mature osteoblasts of adult mice, activated Hh signaling, caused by a deficiency in PTCH1, leads to low bone strength, with reduced bone density attributed to enhanced osteoclast-induced bone resorption [20]. Consistent with this, Hh-signaling inhibition by mature osteoblasts’ specific conditional ablation of *Smo* results in protection from bone loss in one-year-old mice [20]. By contrast, a low level activation of Hh signaling, caused by PTCH1 haploinsufficiency, enhances osteoblast differentiation and increases bone mass [21]. During osteolytic cancer bone metastasis, augmented GLI activity in tumor cells leads to secretion of parathyroid hormone-related protein (PTHrP), which induces the Receptor Activator of Nuclear factor-κB Ligand (RANKL) expression in osteoblasts, thus promoting osteoclastogenesis [22]. These studies have, however, attached importance mainly to the Hh function on osteogenic lineage cells, the specific or direct role of Hh signaling on osteoclastic bone resorption being unknown.

Osteoclasts, differentiated from the monocyte/macrophage lineage stimulated by RANKL, destroy the bone matrix and stimulate osteoblast differentiation and bone formation, thus maintaining bone remodeling [23,24]. Interference of osteoclastic bone resorption is a therapeutic target of anti-osteoporosis drugs, such as bisphosphonates and the anti-RANKL antibody (denosumab) [25,26]. Oral administration of cyclopamine increases bone mass because of the reduced bone resorption in mice, suggesting that cyclopamine can also be a therapeutic drug for osteoporosis [27]. Yet, the mechanism of the inhibitory effect of cyclopamine on bone resorption is not fully understood.

Here, we show that treatment with cyclopamine, GANT-58, or GANT-61 exerts a potent inhibitory effect on osteoclast formation in primary cultured bone marrow-derived macrophages (BMMs) stimulated by RANKL and suggest that Hh signaling is a requisite for osteoclastic differentiation. Moreover, macrophage/osteoclast lineage-specific *Smo* gene deficiency protected from age-related bone loss. Thus, we provide evidence that Hh signaling in the macrophage/osteoclast lineage mediates osteoclastogenesis in vitro and in vivo.

## 2. Results

### 2.1. Changes in Expression of Hedgehog (Hh) Signaling-Related Genes during Osteoclast Differentiation

**Figure 1:**
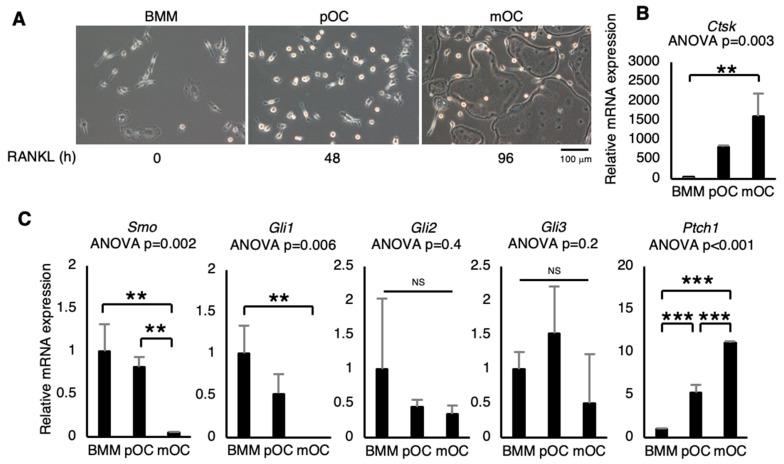
Changes in expression of Hedgehog (Hh) signaling-related genes during osteoclast differentiation. (**A**) Representative microscopic images of bone marrow-derived macrophages (BMMs: without RANKL), pre-osteoclasts (pOC: 48 h after RANKL stimulation), or mature osteoclasts (mOC: 96 h after RANKL stimulation). (**B**) *Ctsk* mRNA expression was determined by quantitative real-time RT-PCR (*n* = 3). (**C**) Hh signaling-related gene expression levels were determined by quantitative real-time RT-PCR (*n* = 3). The abundance of target mRNA was normalized by that of *Actb* mRNA. Data are represented as means ± s.d. One-way ANOVA, followed by Tukey-Kramer’s test; NS, not significant; ** *p* < 0.01; *** *p* < 0.001.

First examined were the changes in the expression of Hh signaling-related genes during osteoclast differentiation. Primary cultured bone marrow-derived macrophages (BMMs) differentiated into mature osteoclasts (mOC) 96 h after RANKL stimulation (Figure 1A). Quantitative real-time RT-PCR (qRT-PCR) analysis revealed that *Ctsk* mRNA expression significantly increased after RANKL stimulation (Figure 1B), suggesting a progressing of osteoclastic differentiation. No obvious change in *Gli2* and *Gli3* mRNA expression, whereas *Smo* and *Gli1* mRNA expression decreased gradually with osteoclastic differentiation, as assessed by qRT-PCR (Figure 1C). On the other hand, *Ptch1* mRNA expression significantly increased gradually with osteoclastic differentiation (Figure 1C). These results suggest that Hh signaling in the macrophage/osteoclast lineage is associated with osteoclastic differentiation.

### 2.2. Cyclopamine Suppresses Osteoclastogenesis

**Figure 2:**
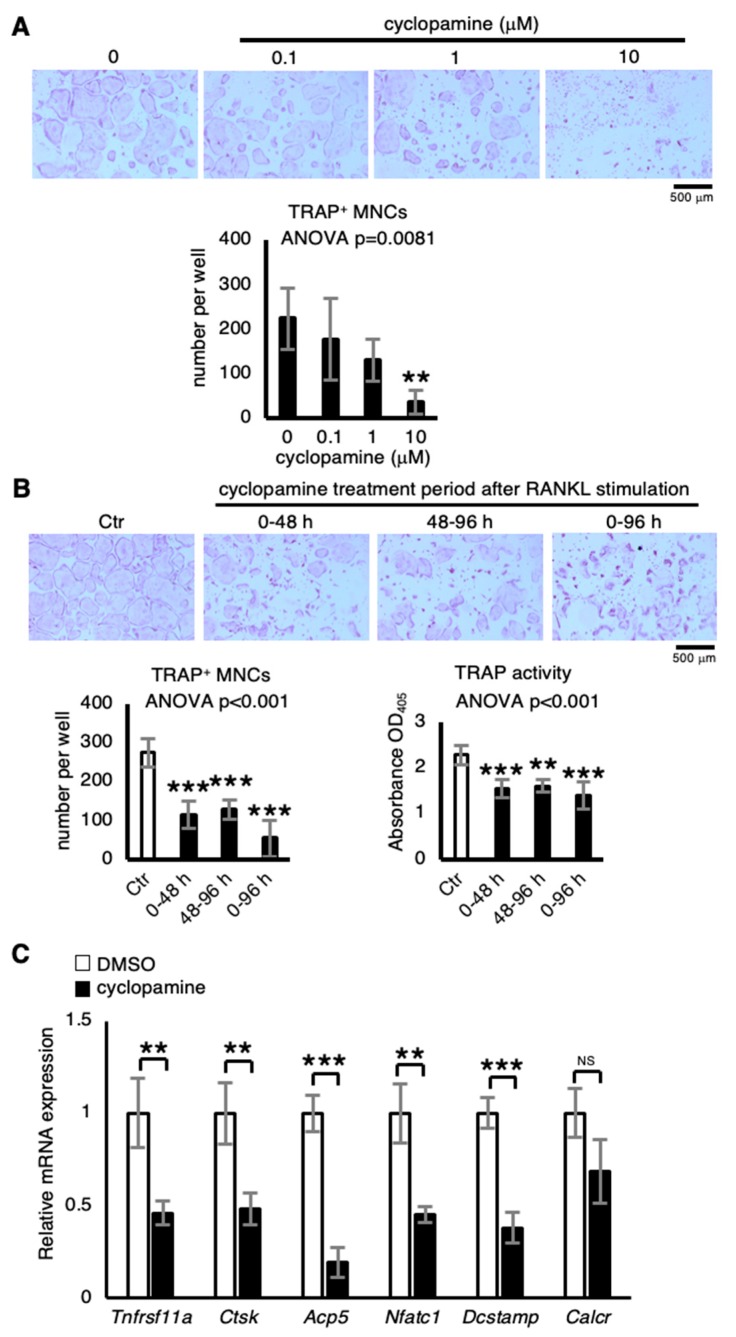
Cyclopamine suppresses osteoclastogenesis. (**A**) The effect of cyclopamine on osteoclast formation. The number of TRAP^+^ multinuclear cells (MNCs) were counted (*n* = 4). (**B**) Osteoclastogenic cultures treated with 10-μM cyclopamine throughout the 96 h period (0–96 h) or only during the first (0–48 h) or second period (48–96 h); the number of TRAP^+^ multinuclear cells (MNCs) and TRAP activity determined 96 h after RANKL stimulation (*n* = 4). (**C**) Effect of cyclopamine treatment on osteoclastic gene expression in osteoclastic cultures (*n* = 3). Abundance of target mRNA normalized by that of *Actb* mRNA. Data are represented as means ± s.d. One-way ANOVA, followed by Dunnett’s test (**A**,**B**) or unpaired two-sided Student’s *t*-test (**C**); NS, not significant; ** *p* < 0.01 and *** *p* < 0.001.

Since treatment with SMO inhibitor cyclopamine increases trabecular bone volume because of reduced bone resorption in mice [27], the effect of cyclopamine treatment on primary osteoclastic cultures was examined. Treatment with cyclopamine significantly decreased formation of TRAP^+^ multinuclear cells (MNCs) in a dose-dependent manner (Figure 2A). Although no recombinant Hh ligand proteins were added to the osteoclastic cultures, cyclopamine treatment suppressed osteoclastogenesis. Possibly, therefore, Hh ligands contained in FBS may be important for osteoclast formation in vitro. That the addition of recombinant Sonic hedgehog enhances osteoclast formation in RAW264.7 cells [28] suggests that Hh ligands mediate osteoclastogenesis. Next, to investigate when it suppresses osteoclast differentiation, cyclopamine treatment was implemented over terms of 0-48 h, 48-96 h, and 0-96 h after RANKL stimulation. The outcome disclosed that the suppression was induced during all terms, as assessed by TRAP staining and a TRAP activity assay (Figure 2B). Moreover, quantitative real-time RT-PCR analysis revealed that treatment with cyclopamine significantly decreased osteoclastic marker genes *Tnfrsf11a* (encoding RANK), *Ctsk*, *Acp5* (encoding TRAP), *Nfatc1*, and *Dcstamp* without affecting *Calcr* (encoding Calcitonin receptor) at 96 h after RANKL stimulation (Figure 2C). These results suggest that cyclopamine induces inhibitory effects at all stages of osteoclastic differentiation.

### 2.3. Other Hh Signaling Inhibitors that Suppress Osteoclastogenesis

**Figure 3:**
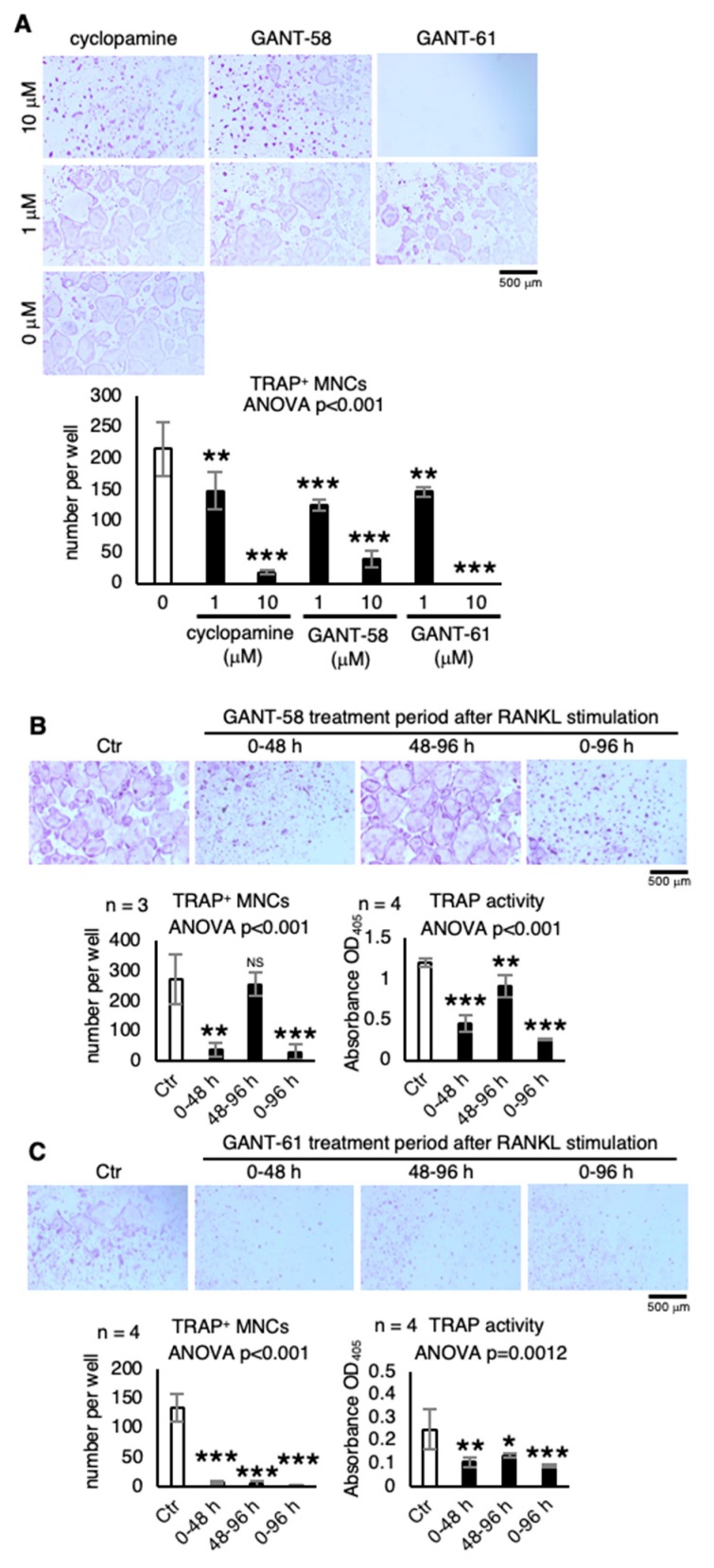
Other Hh signaling inhibitors that suppress osteoclastogenesis. (**A**) Effect of Hh signaling inhibitors cyclopamine, GANT-58, and GANT-61 on osteoclast formation. The number of TRAP^+^ multinuclear cells (MNCs) were counted (*n* = 3). (**B**,**C**) Osteoclastogenic cultures treated with (**B**) 10-μM GANT-58 or (**C**) 5-μM GANT-61 throughout the 96-h period (0–96 h) or only during the first (0–48 h) or second period (48–96 h); the number of TRAP^+^ multinuclear cells (MNCs) and TRAP activity determined at 96 h after RANKL stimulation (*n* = 3–4). Data are represented as means ± s.d. One-way ANOVA, followed by Dunnett’s test; NS, not significant; * *p* < 0.05, ** *p* < 0.01, and *** *p* < 0.001.

Next examined was the effect of other Hh signaling inhibitors, GANT-58 (GLI1 inhibitor) and GANT-61 (GLI1/2 inhibitor), on osteoclast differentiation. Treatment with GANT-58 and GANT-61 significantly decreased TRAP^+^ MNC formation in a dose-dependent manner, as did the treatment with cyclopamine (Figure 3A), strongly suggesting that Hh signaling in the macrophage/osteoclast lineage is requisite for osteoclast formation. Interestingly, GANT-58 treatment during the first 48-h term (0–48 h) was sufficient to inhibit osteoclastogenesis but not during the second term (48–96 h) (Figure 3B). On the other hand, GANT-61 treatment exerted an inhibitory effect on osteoclast differentiation during the three terms, as did cyclopamine treatment (Figure 3C). These results suggest that GLI1 and GLI2 activation is essential for the early and late stages, respectively, of osteoclast differentiation.

### 2.4. Hh Signaling Inhibitors Suppress Osteoclastic Viability

**Figure 4:**
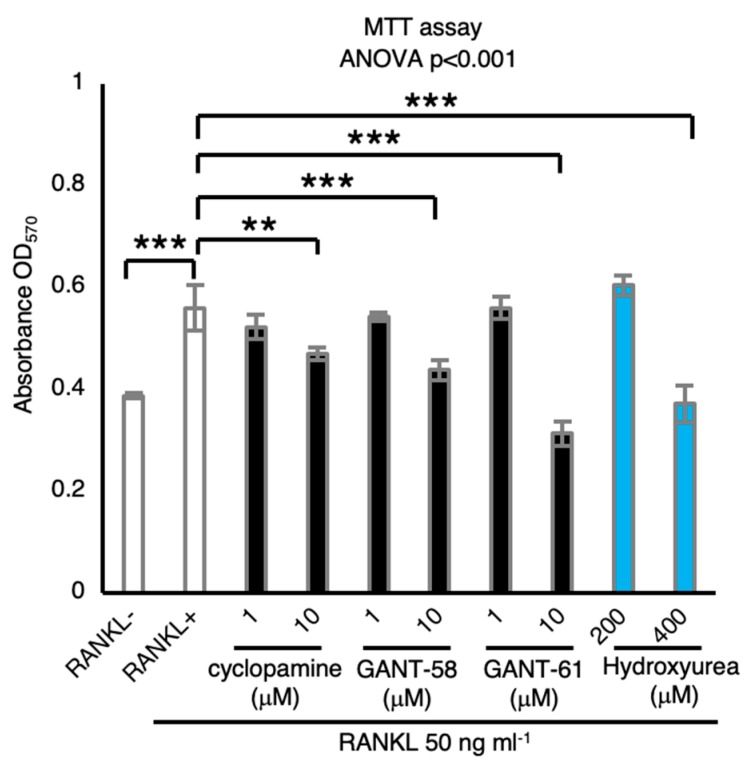
Hh signaling inhibitors suppress osteoclastic viability. The effect of Hh signaling inhibitors on osteoclast viability determined by MTT assay (*n* = 3). Hydroxyurea used as DNA synthesis inhibitor. Data are represented as means ± s.d. One-way ANOVA, followed by Dunnett’s test; NS, not significant; ** *p* < 0.01 and *** *p* < 0.001.

MTT cell viability assay carried out to investigate the effect of Hh signaling inhibitors on osteoclast growth revealed that RANKL stimulation increased the number of BMMs and that Hh inhibitors cyclopamine, GANT-58, and GANT-61 inhibited osteoclastic viability (Figure 4), suggesting that Hh signaling mediates osteoclastic viability.

### 2.5. Macrophage/osteoclast Lineage-Specific Smo Knockout Mice Exhibit Resistance to Age-Related Bone Loss

**Figure 5:**
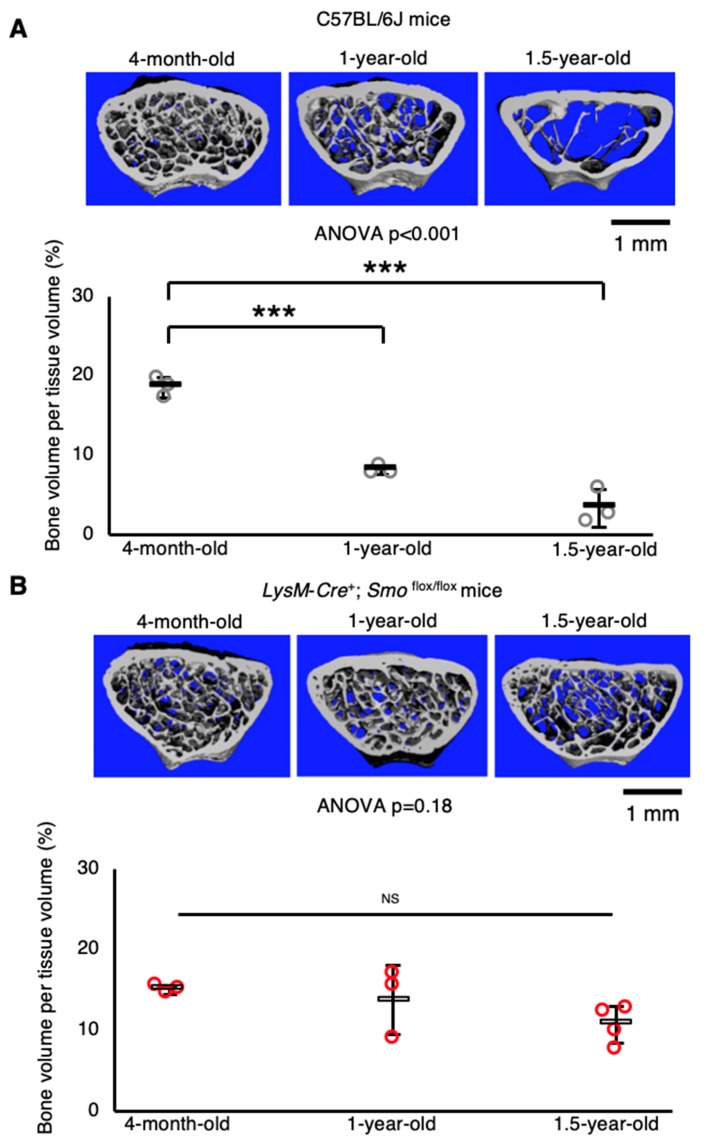
Macrophage/osteoclast lineage-specific *Smo* knockout mice exhibit resistance to age-related bone loss. (**A**,**B**) Distal femurs from (**A**) C57BL/6J male mice or (**B**) *LysM-Cre*^+^; *Smo* ^flox/flox^ male mice scanned in a Scanco Medical μCT 35 System (SCANCO Medical); representative images and three-dimensional bone volume per tissue volume (%) (*n* = 3–4). Data are represented as means ± s.d. One-way ANOVA, followed by Dunnett’s test; NS, not significant, and *** *p* < 0.001. Note that we separated C57BL/6J mice and *LysM-Cre*^+^; *Smo* ^flox/flox^ mice datasets, because we maintained C57BL/6J mice and *LysM-Cre*^+^; *Smo* ^flox/flox^ mice separately.

Finally, to examine the function of Hh signaling in osteoclasts in vivo, macrophage/osteoclast lineage-specific *Smo* knockout mice were generated by crossing *LysM-Cre* mice [29] with *Smo*-floxed mice [30]. A preliminary microcomputed tomography (μCT) analysis revealed that trabecular bone mass decreased significantly with aging in wild-type C57BL/6J mice (Figure 5A). On the other hand, deletion of *Smo* in the macrophage/osteoclast lineage impaired age-related bone loss (Figure 5B), suggesting that Hh signaling in this lineage is associated with bone reduction with aging.

## 3. Discussion

**Figure 6:**
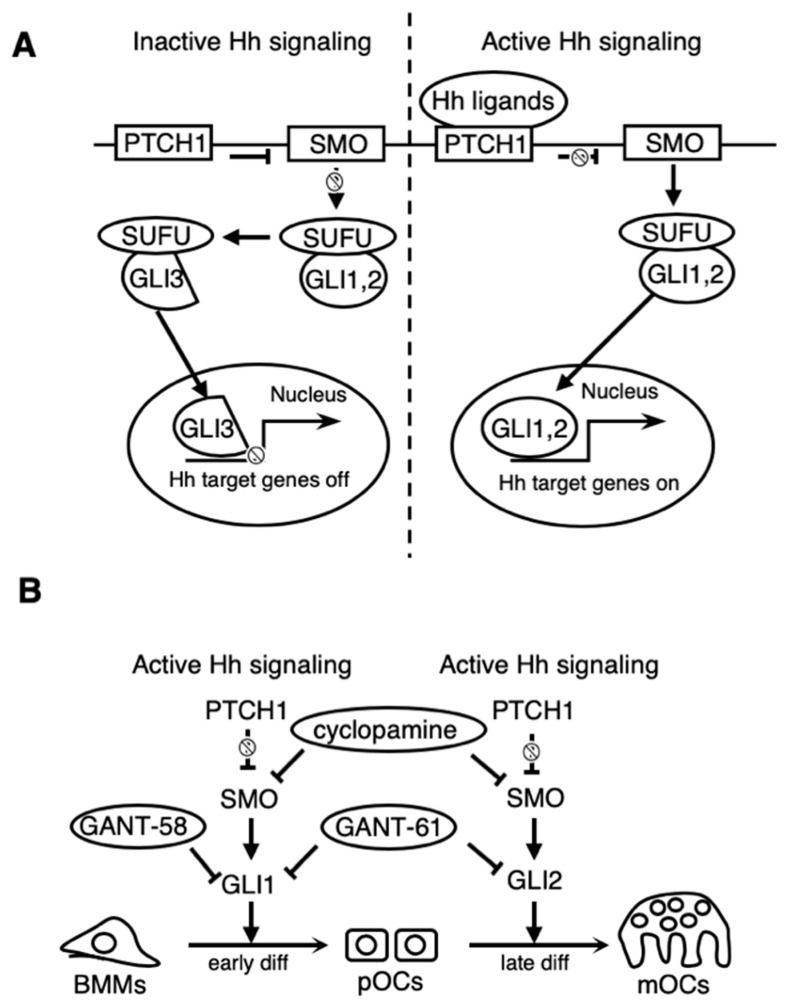
Schema of Hh signaling-mediated osteoclastic differentiation. (**A**) Schematic of the general Hh-signaling pathway. (**B**) Proposed model of the newly identified inhibitory mechanism on osteoclastic differentiation by Hh inhibitors. GLI1 activation is a requisite for the differentiation of bone marrow-derived macrophages (BMMs) into pre-osteoclasts (pOCs). GLI2 activation is a requisite for the differentiation of pOCs into mature osteoclasts (mOCs).

This study demonstrates that Hh inhibitors directly function as suppressors of osteoclast formation in in vitro cultures, indicating that Hh signaling in the macrophage/osteoclast lineage is indispensable for osteoclastogenesis (Figure 6A,B). Interestingly, *Gli1* mRNA expression, an important indicator of Hh signaling [8], dynamically decreased during the differentiation of pre-osteoclasts (pOCs) into mature osteoclasts (mOCs) (Figure 1C). Additionally, treatment with the GLI1 inhibitor GANT-58 during differentiation of pOCs into mOCs did not affect osteoclast formation in vitro (Figure 3B). These findings indicate that GLI1 is not required for the differentiation of pOCs into mOCs. Taken together with a previous study that oral treatment with cyclopamine suppresses osteoclastic function and results in increased bone mass in mice [27], our findings suggest that Hh signaling inhibitors have a potential as anti-resorptive agents.

Osteoporosis is a major problem in public health because of its high morbidity and detriment to the quality of life [31]. As aging can be a risk factor in the development of osteoporosis regardless of gender [32], the mechanisms of age-related bone loss are pertinent to the pathogenesis of osteoporosis. Here, our preliminary μCT analysis demonstrated that macrophage/osteoclast lineage-specific deletion of the *Smo* gene prevents age-related bone loss. As all Hh signaling is transmitted through transmembrane protein SMO, *Smo* deficiency impairs the transmission of all Hh signaling [33]. Thus, we provide evidence that Hh signaling in the macrophage/osteoclast lineage is a regulator of age-related bone loss. Ablation of senescent cells pharmacologically or genetically in mice ameliorates age-related bone loss, suggesting that senescence-associated secretory phenotype (SASP) proteins, secreted by senescent cells, account for senile bone loss [34]. Possibly, therefore, Hh ligands may be a kind of SASP protein; nevertheless, no such evidence has been available to date. Since no other hypotheses have been tested, further comprehensive studies are needed to elucidate relevant underlying mechanisms.

In conclusion, this study demonstrates that Hh signaling in the macrophage/osteoclast lineage is requisite for osteoclastogenesis in in vitro cultures and that it mediates age-related bone loss in vivo, indicating the therapeutic potential of Hh inhibitors for senile bone loss.

## 4. Materials and Methods

### 4.1. Reagents 

Recombinant M-CSF protein (Cat# 416-ML-500) was purchased from R&D Systems, Minneapolis, MN, USA; GST-RANKL (Cat# 47197900) from Oriental Yeast, Tokyo, Japan; cyclopamine (SMO inhibitor, Cat# 038-19311) from Wako, Osaka, Japan; GANT-58 (GLI1 inhibitor, Cat# CS-0507) from Chem Scene, Monmouth, NJ, USA; and GANT-61 (GLI1/2 inhibitor, Cat# AG-CR1-3561) from AdipoGen, Seoul, Korea. 

### 4.2. Mice 

C57BL/6J mice were purchased from Clea, Tokyo, Japan. *Smo*-floxed mouse [30] and *LsyM-Cre* mouse strains [29] were obtained from Jackson Laboratory, Bar Harbor, ME, USA. All strains were on a C57BL/6J background. All mice were maintained in a specific pathogen-free facility under climate-controlled conditions and a 12-h light/dark cycle and provided with water and standard diet (Oriental Yeast) ad libitum. All animals were handled according to the protocol approved by the Animal Experiment Committee of Ehime University, Japan (Permit No. 05-KU-36-16).

### 4.3. Generating M-CSF Overexpression Cells

To obtain M-CSF overexpression cells, mouse M-CSF cDNA in the pCAG-Neo mIgG2a-Fc vector (Wako) was transfected into mouse fibroblastic LMTK^−^ cells (ATCC, Cat# CCL-1.3) with X-tremeGENE HP DNA transfection reagent (Roche Diagnostics, Basle, Switzerland). After selection with 5-mg mL^−1^ G418 (Wako), clones producing high M-CSF-Fc levels were selected by limiting dilution followed by ELISA for Fc-tag protein expression, and the cell culture supernatant of the highest M-CSF-Fc-producing cell line, LMF44-11, was harvested.

### 4.4. Osteoclast Culture

For in vitro osteoclast formation, whole bone marrow cells were harvested from tibias and femurs of eight ten-week-old mice, cultured for three days in α-MEM containing antibiotic-antimycotic solution (Gibco, Grand Island, NY, USA) and 10% FBS supplemented with LMF44-11-conditioned medium used as a source of M-CSF to obtain bone marrow-derived macrophages (BMMs). BMMs were further cultured for four days in medium supplemented with 50-ng mL^−1^ M-CSF and 50-ng mL^−1^ RANKL. The culture medium was changed every second day. Osteoclasts were identified by TRAP staining or TRAP activity assay, as described in [35,36].

### 4.5. RNA Isolation and Real-Time RT-PCR 

RNA isolation and real-time RT-PCR were carried out as described in [35,36]. Briefly, total RNA was extracted using TRIzol reagent (Invitrogen, Carlsbad, CA, USA); cDNA was transcribed and used for quantitative RT-PCR conducted with Thunderbird SYBR qPCR Mix (Toyobo Co., Ltd., Osaka, Japan) in the 7500 fast real-time PCR system (Applied Biosystems, Carlsbad, CA, USA). The primers used for RT-PCR are shown in Table S1.

### 4.6. MTT Assay 

MTT (3-(4, 5-Dimethylthial-2-yl)-2, 5-Diphenyltetrazalium Bromide) assay was performed as described in [27]. Briefly, BMMs (5 × 10^3^ per well) were plated in 96-well plates with indicated drugs. After 48 h, MTT (Wako) was added for 4 h. HCl/isopropanol was added to measure absorbance at 570 nm. 

### 4.7. Microcomputed Tomography

Microcomputed tomography (μCT) scanning of the distal femurs was carried out in a Scanco Medical μCT 35 System (SCANCO Medical). 

### 4.8. Statistical Analysis 

All data are expressed as the means ± s.d. Statistical analyses were carried out with one-way ANOVA followed by Tukey-Kramer’s test or Dunnett’s test or unpaired two-sided Student’s *t*-test (* *p* < 0.05, ** *p* < 0.01, and *** *p* < 0.001; NS, not significant).

## Supplementary Materials

Supplementary materials can be found at https://www.mdpi.com/1422-0067/21/8/2745/s1.
