# Supplementary material for: Hedgehog Inhibitors Suppress Osteoclastogenesis in In Vitro Cultures, and Deletion of Smo in Macrophage/Osteoclast Lineage Prevents Age-Related Bone Loss
Source: Int J Mol Sci. 2020 Apr 15;21(8):2745. doi: 10.3390/ijms21082745 (PMC7216259; doi:10.3390/ijms21082745)
Supplement: Supplementary file 1 [file ijms-21-02745-s001.pdf]

Supplementary material

Article

Hedgehog Inhibitors Suppress Osteoclastogenesis in in Vitro Cultures, and Deletion of *Smo* in Macrophage/Osteoclast Lineage Prevents Age-Related Bone Loss

Yukihiro Kohara[1,*], Ryuma Haraguchi[1,*], Riko Kitazawa [1,2], Yuuki Imai [3,4,5] and Sohei Kitazawa [1].

1 Department of Molecular Pathology, Ehime University Graduate School of Medicine, Shitsukawa, Toon City, Ehime 791-0295, Japan; riko@m.ehime-u.ac.jp (R.K.); kitazawa@m.ehime-u.ac.jp (S.K.)
2 Division of Diagnostic Pathology, Ehime University Hospital, Shitsukawa, Toon City, Ehime 791-0295, Japan
3 Division of Integrative Pathophysiology, Proteo-Science Center, Ehime University Graduate School of Medicine, Shitsukawa, Toon City, Ehime 791-0295, Japan
4 Division of Laboratory Animal Research, Advanced Research Support Center, Ehime University, Toon, Ehime 791-0295, Japan
5 Department of Pathophysiology, Ehime University Graduate School of Medicine, Toon, Ehime 791-0295, Japan
* Correspondence: kohara.yukihiro.yu@ehime-u.ac.jp (Y.K.); ryumaha@m.ehime-u.ac.jp (R.H.);

Received: 24 March 2020; Accepted: 14 April 2020; Published: date

Supplementary Table 1: Oligonucleotide primers for quantitative RT-PCR

Gene	Forward primer (5'-3')	Reverse primer (5'-3')
Actb	AAGGCCAACCGTGAAAAGAT	GTGGTACGACCAGAGGCATAC
Smo	GGGATCCATTCATTCCCGCA	GTGCTCTCAGGAAGAGCCAT
Gli1	GACGGAGGTCTCTTTGTCCG	CTCAGGGAAGGATGAGGGGA
Gli2	GCCTGAATAGACTGTGGGTGA	ACTGTCAAGGGAAAAGCAGATCA
Gli3	AATGCTTCAGATCGGGCCAA	CTGGGGTCTGTGTAACGCTT
Tnfrsf11a	TCGTCCACAGACAAATGCAAA	GTGTGCTTCTAGCTTTCCAAGG
Ctsk	CTCCATCGACTATCGAAAGAAAG	AAAGCCCAACAGGAACCAC
Acp5	CGTCTCTGCACAGATTGCAT	AAGCGCAAACGGTAGTAAGG
Nfatc1	CTCGAAAGACAGCACTGGA	ACCGACAGATACTGCTCGCAAA
Dcstamp	CGTGGGCCAGAAGTTGCT	AGGCCAGTGCTGACTAGGATGA
Calcr	CCTTCCAGAGGAGAAGAAACC	GGAGATTCCGCCTTTTCAC
